# Palmitoylation-related lncRNAs link molecular signaling, immune remodeling, and tumor progression in glioma: prognostic modeling and functional validation of LYRM4-AS1

**DOI:** 10.3389/fnmol.2026.1901839

**Published:** 2026-09-10

**Authors:** Juan Xu, Chen Zhang, Lingling Pu, Xiaoyang Zhu, Xiaoan Sheng, Juanjuan Dong, Miaomiao Song, Chao Wang

**Affiliations:** 1 Department of Oncology, The Fourth Affiliated Hospital of Anhui Medical University, Hefei, China; 2 Hubei University of Medicine, Shiyan, Hubei, China; 3 Department of Encephalopathy, The Second Affiliated Hospital of Anhui University of Chinese Medicine, Hefei, China

**Keywords:** glioma, immune microenvironment, long non-coding RNA, LYRM4-AS1, molecular signaling, neuro-oncology, palmitoylation, TIDE

## Abstract

**Background:**

Glioma is a molecularly heterogeneous tumor of the central nervous system with variable clinical behavior, immune contexture, and therapeutic response. Palmitoylation is a reversible lipid modification that regulates membrane localization, synaptic signaling, inflammatory pathways, and oncogenic signaling; however, the prognostic and functional relevance of palmitoylation-related long non-coding RNAs (PRlncRNAs) in glioma remains incompletely defined.

**Methods:**

Transcriptomic and clinical data for 429 glioma patients were obtained from TCGA. Thirty palmitoylation-related genes were used to identify PRlncRNAs through co-expression analysis. A prognostic signature was constructed using univariate Cox regression, LASSO-Cox regression, and multivariate Cox regression. The model was evaluated by Kaplan-Meier analysis, ROC curves, time-dependent ROC analysis, Cox regression, C-index analysis, nomogram modeling, calibration curves, and an institutional clinical cohort of 46 paired glioma and adjacent tissues. Functional enrichment, immune deconvolution, tumor microenvironment scoring, TIDE-based immunotherapy prediction, TMB/MSI analysis, and pRRophetic-based drug-sensitivity analysis were performed. U87 and U251 glioma cells, LYRM4-AS1 knockdown, *in vitro* functional assays, and nude-mouse xenografts were used for biological validation.

**Results:**

A six-PRlncRNA signature consisting of POLR2J4, LYRM4-AS1, BX640514.2, AL592295.6, AL390755.1, and AC018730.1 stratified glioma patients into high- and low-risk groups with significantly different overall survival. The risk score remained an independent prognostic factor and showed strong predictive performance for 1-, 3-, and 5-year survival. Risk-associated genes were enriched in extracellular matrix organization, leukocyte-mediated immunity, neuroactive ligand-receptor interaction, MAPK signaling, cytokine-receptor interaction, cell-cycle programs, and ECM-receptor interaction. High-risk tumors displayed increased stromal, immune, and ESTIMATE scores, higher expression of multiple immune checkpoint genes, higher TIDE scores, and elevated TMB/MSI levels. Clinical validation confirmed differential expression of the six PRlncRNAs and the prognostic value of the risk score. Functionally, LYRM4-AS1 knockdown inhibited glioma cell proliferation, EdU incorporation, migration, invasion, and colony formation, while suppressing xenograft growth, increasing TUNEL-positive apoptotic cells, and reducing Ki-67, CD31, and MMP9 expression.

**Conclusion:**

This study identifies a palmitoylation-related lncRNA signature that integrates molecular signaling, immune remodeling, and prognosis in glioma. LYRM4-AS1 acts as a functional driver of glioma progression and may represent a candidate biomarker and therapeutic target at the crossroads of neural tumorigenesis and immune-microenvironmental regulation.

## 1 Introduction

Gliomas are the most common primary malignant tumors of the central nervous system and are characterized by marked molecular, histological, and clinical heterogeneity (Ostrom et al., 2022). Current classification integrates histopathology with molecular features, including IDH mutation, 1p/19q co-deletion, and MGMT promoter methylation (Louis et al., 2021). These biomarkers strongly influence prognosis and therapeutic decision-making (Wen et al., 2020). Despite maximal safe resection, radiotherapy, temozolomide-based chemotherapy, and emerging molecularly guided strategies, patients with aggressive gliomas, particularly glioblastoma, continue to experience poor long-term survival and frequent recurrence (Stupp et al., 2005). These limitations indicate that additional molecular biomarkers are needed to capture the biological programs that drive glioma progression, immune remodeling, and therapeutic resistance.

Large-scale transcriptomic studies have shown that gliomas are not homogeneous entities but comprise distinct molecular states and cellular ecosystems. TCGA analyses established major transcriptional and genomic subclasses of glioblastoma, linking molecular state to survival and therapeutic response (Brennan et al., 2013). Integrated lower-grade glioma studies further connected genomic alterations with lineage features and clinical outcome (Ceccarelli et al., 2016). Subsequent molecular taxonomy studies refined the understanding of glioma subtype evolution and transcriptional heterogeneity (Verhaak et al., 2010). Single-cell analyses revealed that glioma cells dynamically occupy neural-progenitor-like, oligodendrocyte-progenitor-like, astrocytic, and mesenchymal-like states (Neftel et al., 2019). These cellular-state transitions indicate that malignant cells can exploit developmental and regenerative transcriptional programs (Patel et al., 2014). Glioma progression is also shaped by the brain tumor microenvironment, especially microglia/macrophages and lymphocytes (Quail and Joyce, 2017). Stromal components, vascular niches, and inflammatory signaling networks further contribute to invasion, immune escape, and therapeutic resistance (Hambardzumyan et al., 2016). These interactions support the concept that glioma biology emerges from coordinated tumor-cell and microenvironmental programs (Klemm et al., 2020). This perspective is consistent with the broader framework in which neural repair, degeneration, and tumorigenesis share partially overlapping molecular circuits (Friebel et al., 2020).

Protein palmitoylation is a reversible post-translational lipid modification that attaches fatty acyl chains to proteins and regulates membrane association, trafficking, stability, and signaling activity (Linder and Deschenes, 2007). Palmitoylation is mediated by DHHC family palmitoyltransferases and reversed by depalmitoylating enzymes, allowing dynamic control of protein localization and function (Resh, 2016). Curated palmitoylation resources and enzymology studies have provided a basis for systematic interrogation of palmitoylation-related genes (Chamberlain and Shipston, 2015). In the nervous system, palmitoylation is particularly relevant because it controls synaptic localization, receptor trafficking, neuronal excitability, and activity-dependent plasticity (Fukata and Fukata, 2010). Dysregulated palmitoylation can therefore affect both neural function and malignant transformation. Pan-cancer palmitoylation profiling has suggested that palmitoylation-related genes participate in tumorigenesis and immune regulation (Kong et al., 2023). In hepatocellular carcinoma, palmitoylation-related gene signatures have been linked to prognosis, immune infiltration, and therapeutic vulnerability (Feng et al., 2024). However, the non-coding RNA layer associated with palmitoylation in glioma remains poorly characterized.

Long non-coding RNAs (lncRNAs) regulate gene expression through chromatin remodeling and transcriptional regulation (Schmitt and Chang, 2016). They also participate in RNA stability, competing endogenous RNA networks, and interactions with RNA-binding proteins (Statello et al., 2021). In cancer, lncRNAs contribute to proliferation, invasion, stemness, immune evasion, and treatment resistance (Bhan et al., 2017). Their tissue-specific expression and regulatory diversity make them attractive candidates for tumor biomarker discovery (Ransohoff et al., 2018). Because lncRNAs can integrate molecular signaling states with tumor-cell behavior and microenvironmental responses, palmitoylation-related lncRNAs may provide a biologically meaningful strategy for glioma risk stratification and mechanistic discovery.

Pathway-oriented transcriptomic modeling has become increasingly useful for identifying prognostic and immunotherapy-related biomarkers. In sarcoma, EXT2 was connected to tumor progression and immune evasion through the AKT/c-Myc/PD-L1 axis, providing evidence that multi-omics modeling can uncover actionable immune-oncogenic signaling (Qin et al., 2026b). Ubiquitination-related signatures have been used to stratify prognosis and immunotherapy response in sarcoma (Qin et al., 2024a). Disulfidptosis-related signatures have also shown prognostic and immunotherapy-related value in sarcoma (Xu et al., 2024). m7G-related biomarkers in sarcoma support the value of mechanism-centered molecular signatures (Qin et al., 2023a). Lysine crotonylation-related lncRNAs in glioma further illustrate the relevance of epigenetic-state-associated non-coding RNA models (Song et al., 2025). In hepatocellular carcinoma, migrasome-related lncRNAs have been linked to prognosis and immune response (Qin et al., 2025a). Cuproptosis-related gene signatures have also been used to characterize the immune microenvironment in hepatocellular carcinoma (Qin et al., 2023b). In lung adenocarcinoma, anoikis-related models have been integrated with immunotherapy prediction and oncogene validation (Qin et al., 2024b). Disulfidptosis-related signatures in head and neck squamous cell carcinoma provide another example of pathway-specific biomarker development (Qin et al., 2025b). m7G methylation-associated genes have similarly been linked to prognosis and immunotherapy response in head and neck squamous cell carcinoma (Xu et al., 2025). These studies collectively support the use of mechanism-centered transcriptomic modeling in glioma.

## 2 Materials and methods

### 2.1 Data acquisition and preprocessing

RNA-sequencing expression profiles and clinical information for glioma patients were obtained from The Cancer Genome Atlas (TCGA) database (Tomczak et al., 2015). A total of 429 glioma patients with available transcriptomic and survival information were included. Expression values were processed as transcripts per million (TPM) and log2 transformed where appropriate before downstream analyses. Thirty palmitoylation-related genes (PRGs) were curated from previous palmitoylation studies and are listed in Supplementary Table S1. LncRNA and mRNA expression matrices were extracted from the TCGA-Glioma transcriptomic dataset and matched with clinical data.

### 2.2 Identification of palmitoylation-related lncRNAs and construction of the prognostic signature

Pearson correlation analysis was performed between PRGs and lncRNAs to identify palmitoylation-related lncRNAs (PRlncRNAs). LncRNAs with an absolute correlation coefficient > 0.4 and *P* < 0.001 were defined as PRlncRNAs. Univariate Cox regression was then used to identify survival-related PRlncRNAs, and PRlncRNAs with *P* < 0.05 were retained. LASSO regression was performed using the glmnet package with 10-fold cross-validation to reduce overfitting and refine the candidate variables (Friedman et al., 2010). Cox-model regularization was implemented according to established procedures for penalized Cox regression (Simon et al., 2011). Multivariate Cox regression was subsequently used to construct the final prognostic model. The risk score was calculated as follows: risk score = sum(coefficient of each PRlncRNA × expression level of each PRlncRNA). Patients were divided into high- and low-risk groups according to the median risk score.

### 2.3 Prognostic validation of the risk model

The TCGA-Glioma cohort was randomly divided into training and validation cohorts. Risk-score distribution plots, survival-status plots, expression heatmaps, Kaplan-Meier survival curves, and log-rank tests were used to evaluate the prognostic value of the signature in the total, training, and validation cohorts. Time-dependent ROC curves were generated to evaluate 1-, 3-, and 5-year survival prediction, and ROC analysis was performed using the pROC and timeROC packages where appropriate (Robin et al., 2011). Clinicopathological data, including age, sex, tumor grade, IDH status, 1p/19q co-deletion status, MGMT promoter methylation status, overall survival time, and survival status, were obtained from TCGA-Glioma and are summarized in Supplementary Table S2. The distribution of clinical features between high- and low-risk groups was visualized using ComplexHeatmap. Subgroup survival analyses were performed according to clinically relevant variables.

### 2.4 Cox regression, nomogram construction, and decision curve analysis

Univariate and multivariate Cox proportional hazards regression analyses were performed to determine whether the risk score was an independent prognostic factor. A nomogram integrating the risk score and clinically relevant prognostic factors was constructed to predict 1-, 3-, and 5-year overall survival. Calibration curves were used to compare predicted survival probability with observed survival outcomes. The concordance index (C-index) was used to compare predictive performance over time. Decision curve analysis (DCA) was used to estimate the clinical net benefit of the risk-score model (Vickers and Elkin, 2006).

### 2.5 Functional enrichment analysis

Differentially expressed genes (DEGs) between the high- and low-risk groups were identified using the limma package (Ritchie et al., 2015). Genes with adjusted *P* < 0.05 and |log2 fold change| ≥ 1 were considered DEGs. The resulting DEG set is provided in Supplementary Table S4. Gene Ontology (GO) and Kyoto Encyclopedia of Genes and Genomes (KEGG) enrichment analyses were conducted using clusterProfiler (Yu et al., 2012). Gene set enrichment analysis (GSEA) was performed to identify risk-associated signaling programs and biological pathways (Subramanian et al., 2005).

### 2.6 Immune infiltration and tumor microenvironment analysis

CIBERSORT was used to estimate the relative abundance of immune-cell subsets in each glioma sample (Newman et al., 2015). Single-sample gene set enrichment analysis (ssGSEA) was performed using the GSVA package to quantify immune-cell and immune-function enrichment scores (Hanzelmann et al., 2013). The xCell algorithm was used to infer tissue cellular heterogeneity (Aran et al., 2017). quanTIseq was applied as an additional immune deconvolution method (Finotello et al., 2019). TIMER was used to provide complementary tumor-infiltrating immune-cell estimates (Li et al., 2017). EPIC was included to enumerate cancer and immune cell populations from bulk transcriptomes (Racle et al., 2017). MCP-counter was used as a further method for estimating immune and stromal cell abundance (Becht et al., 2016). StromalScore, ImmuneScore, and ESTIMATEScore were calculated using the ESTIMATE algorithm to evaluate tumor microenvironmental composition (Yoshihara et al., 2013). Correlations between the risk score and tumor microenvironment scores were assessed using Spearman correlation analysis.

### 2.7 Immunotherapy response, TMB/MSI, and drug-sensitivity analyses

Immune checkpoint genes, including CD274, CTLA4, HAVCR2, LAG3, PDCD1, PDCD1LG2, TIGIT, SIGLEC15, ITPRIPL1, and IGSF8, were compared between high- and low-risk groups. Spearman correlation analysis was used to assess associations between the risk score and checkpoint gene expression. The Tumor Immune Dysfunction and Exclusion (TIDE) algorithm was used to estimate immunotherapy response, T-cell dysfunction, and T-cell exclusion scores (Jiang et al., 2018). The IMvigor210 anti-PD-L1 cohort was used as an external exploratory immunotherapy dataset (Mariathasan et al., 2018). Tumor mutation burden (TMB) and microsatellite instability (MSI) scores were compared between risk groups, and combined survival analyses were performed according to TMB/MSI status and risk classification. Drug sensitivity was predicted using the pRRophetic package (Geeleher et al., 2014). The Genomics of Drug Sensitivity in Cancer resource provided a pharmacogenomic basis for drug-response estimation (Yang et al., 2013). Large-scale pharmacogenomic evidence was used to support interpretation of transcriptome-drug response relationships (Iorio et al., 2016). Predicted half-maximal inhibitory concentration (IC50) values were compared between high- and low-risk groups using the Wilcoxon rank-sum test.

### 2.8 Human specimens and clinical validation

Forty-six paired glioma tissues and adjacent non-tumor tissues were obtained from the Fourth Affiliated Hospital of Anhui Medical University (Supplementary Table S3). For the immunotherapy-response validation shown in Figure 6H, an institutional self-collected anti-PD-1/PD-L1-treated cohort with available expression and clinical response data was analyzed (CR/PR, *n = 16*; PD/SD, *n = 30*). Patients were classified according to the original clinical response records. All samples were formalin-fixed, paraffin-embedded, and histologically confirmed by experienced pathologists. Follow-up information was collected for survival analysis. The study was approved by the Ethics Committee of the Fourth Affiliated Hospital of Anhui Medical University (approval No. KYXM-202509-006), and written informed consent was obtained from all patients. qRT-PCR was used to detect the expression of POLR2J4, LYRM4-AS1, BX640514.2, AL592295.6, AL390755.1, and AC018730.1 in paired clinical tissues. The risk score was calculated using the formula derived from the TCGA-Glioma cohort and used to stratify patients for clinical validation.

### 2.9 Cell culture and transfection

Human glioma cell lines U87, U251, and LN229 and normal human astrocytes (NHA) were used for experimental validation. U87 cells were obtained from ATCC (HTB-14), U251 cells were obtained from a certified cell bank, LN229 cells were obtained from ATCC (CRL-2611), and NHA cells were obtained from ScienCell Research Laboratories. Cells were maintained in Dulbecco’s modified Eagle’s medium (DMEM; Gibco, C11995500BT) supplemented with 10% fetal bovine serum (FBS; Gibco, 10099141C) and 1% penicillin-streptomycin (Gibco, 15140122) at 37 °C in a humidified incubator containing 5% CO_2_. All cell lines were authenticated by short tandem repeat profiling and tested negative for mycoplasma contamination before use. Three independent short-hairpin RNAs targeting LYRM4-AS1 and a non-targeting shRNA control were synthesized by GenePharma (Shanghai, China). For *in vitro* transient knockdown experiments, U87 and U251 cells were transfected with sh-LYRM4-AS1 or sh-NC using Lipofectamine 3000 (Invitrogen, L3000015) according to the manufacturer’s protocol. Knockdown efficiency was assessed by qRT-PCR 48 h after transfection, and the most efficient shRNA was selected for subsequent functional assays. For xenograft experiments, U87 cells stably expressing sh-NC or sh-LYRM4-AS1 were generated by lentiviral infection followed by puromycin selection. Briefly, U87 cells were infected with lentiviral particles carrying sh-NC or sh-LYRM4-AS1 at an appropriate multiplicity of infection in the presence of polybrene. After infection, cells were selected with puromycin to establish stable knockdown cell lines. The knockdown efficiency of stable cells was confirmed by qRT-PCR before subcutaneous implantation.

### 2.10 RNA extraction and qRT-PCR

Total RNA from tissues and cells was extracted using TRIzol Reagent (Invitrogen, 15596026). RNA concentration and purity were measured by absorbance at 260/280 nm. Reverse transcription was performed using the PrimeScript RT reagent Kit with gDNA Eraser (Takara, RR047A). qRT-PCR was performed using TB Green Premix Ex Taq II (Takara, RR820A) on a real-time PCR system. GAPDH was used as the internal control, and qRT-PCR reporting followed the MIQE principle where applicable (Bustin et al., 2009). Relative expression levels were calculated using the 2^∧^−ΔΔCt method (Livak and Schmittgen, 2001). Primers were synthesized by Sangon Biotech (Shanghai, China).

### 2.11 Cell proliferation, EdU, and colony-formation assays

For CCK-8 assays, transfected U87 and U251 cells were seeded into 96-well plates at 2,000 cells per well. Cell viability was measured at 0, 24, 48, and 72 h using Cell Counting Kit-8 (Beyotime, C0038), and absorbance at 450 nm was measured using a microplate reader. EdU incorporation was assessed using the BeyoClick EdU-594 Cell Proliferation Kit (Beyotime, C0078S). Cells were fixed, permeabilized, incubated with EdU reaction mixture, counterstained with DAPI (Invitrogen, D1306), and imaged by fluorescence microscopy. For colony-formation assays, transfected cells were seeded into six-well plates and cultured for 10–14 days. Colonies were fixed with 4% paraformaldehyde (Beyotime, P0099), stained with crystal violet, photographed, and counted.

### 2.12 Wound-healing and transwell assays

For wound-healing assays, transfected cells were cultured to near confluence in six-well plates. A linear scratch was generated using a sterile 200-μL pipette tip, and detached cells were removed with phosphate-buffered saline. Images were captured at 0 and 24 h, and migration rates were quantified using ImageJ (Schneider et al., 2012). For Transwell migration assays, cells suspended in serum-free medium were added to the upper chamber of 24-well Transwell inserts (Corning, 3422), while medium containing 10% FBS was added to the lower chamber. For invasion assays, inserts were precoated with Matrigel (Corning, 354234). Migrated or invaded cells were fixed, stained with crystal violet, photographed, and counted in randomly selected fields.

### 2.13 Xenograft model

BALB/c nude mice were used to evaluate the *in vivo* role of LYRM4-AS1. All animal procedures were approved by the Animal Ethics Committee of Anhui Medical University (approval No. LLSC20242305). U87 cells stably expressing sh-NC or sh-LYRM4-AS1 were suspended in phosphate-buffered saline and injected subcutaneously into nude mice. Mice were randomly assigned to sh-NC and sh-LYRM4-AS1 groups. Tumor length and width were measured every 4 days using calipers, and tumor volume was calculated as volume = length × width^2/2. At the experimental endpoint, tumors were collected, photographed, weighed, and fixed in 4% paraformaldehyde for histological analysis.

### 2.14 TUNEL staining and immunohistochemistry

Apoptotic cells in xenograft tumor sections were detected using the One Step TUNEL Apoptosis Assay Kit (Beyotime, C1086). Paraffin-embedded sections were deparaffinized, rehydrated, treated with proteinase K, and incubated with TUNEL reaction mixture at 37 ° C in the dark. Nuclei were counterstained with DAPI, and images were acquired under a fluorescence microscope. For immunohistochemistry, tumor sections were deparaffinized, rehydrated, and subjected to heat-mediated antigen retrieval using EDTA antigen retrieval solution (Beyotime, P0085). Endogenous peroxidase activity was blocked, followed by serum blocking. Sections were incubated overnight at 4 °C with primary antibodies against Ki-67 (Abcam, ab16667), CD31 (Abcam, ab28364), and MMP9 (Abcam, ab76003). Sections were then incubated with HRP-conjugated goat anti-rabbit IgG (Beyotime, A0208). Immunoreactivity was visualized using a DAB Horseradish Peroxidase Color Development Kit (Beyotime, P0203), followed by hematoxylin counterstaining. Integrated optical density per unit area was quantified using ImageJ.

### 2.15 Statistical analysis

All statistical analyses were performed using R software (version 4.4.2) and GraphPad Prism where appropriate. Continuous variables between two groups were compared using Student’s *t*-test or the Wilcoxon rank-sum test according to data distribution. Survival curves were generated using Kaplan-Meier analysis and compared using the log-rank test. Cox proportional hazards regression was used for univariate and multivariate survival analyses. Correlations were assessed using Pearson or Spearman analysis as appropriate. Experimental data are presented as mean ± standard deviation. *P* < 0.05 was considered statistically significant.

## 3 Results

### 3.1 Identification of prognosis-related PRlncRNAs and construction of the glioma risk signature

**FIGURE 1:**
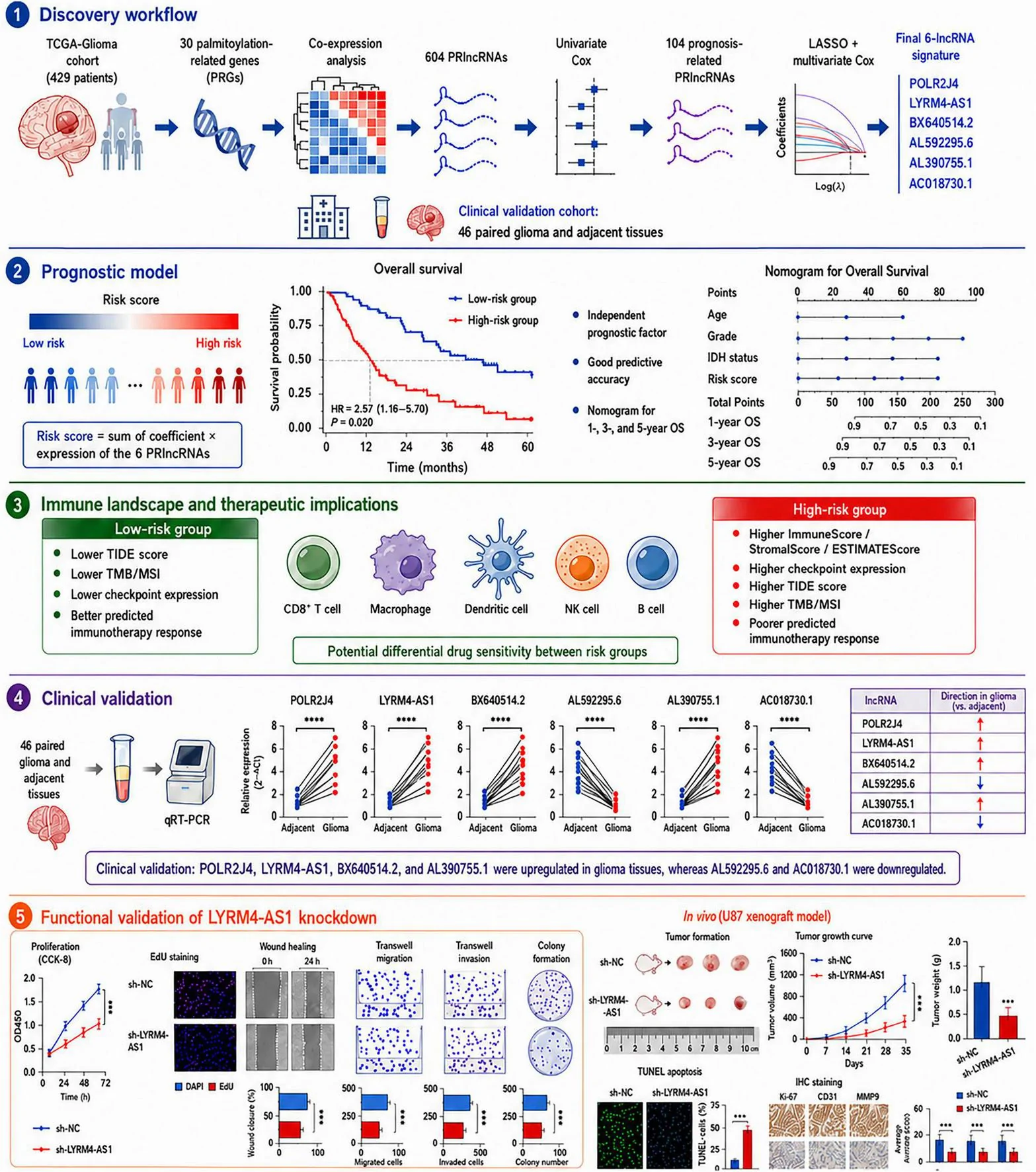
Study workflow. Schematic workflow of the study, including TCGA-Glioma data acquisition, PRG-lncRNA co-expression analysis, prognostic model construction, immune and therapeutic analyses, clinical validation, and functional validation of LYRM4-AS1.

**FIGURE 2:**
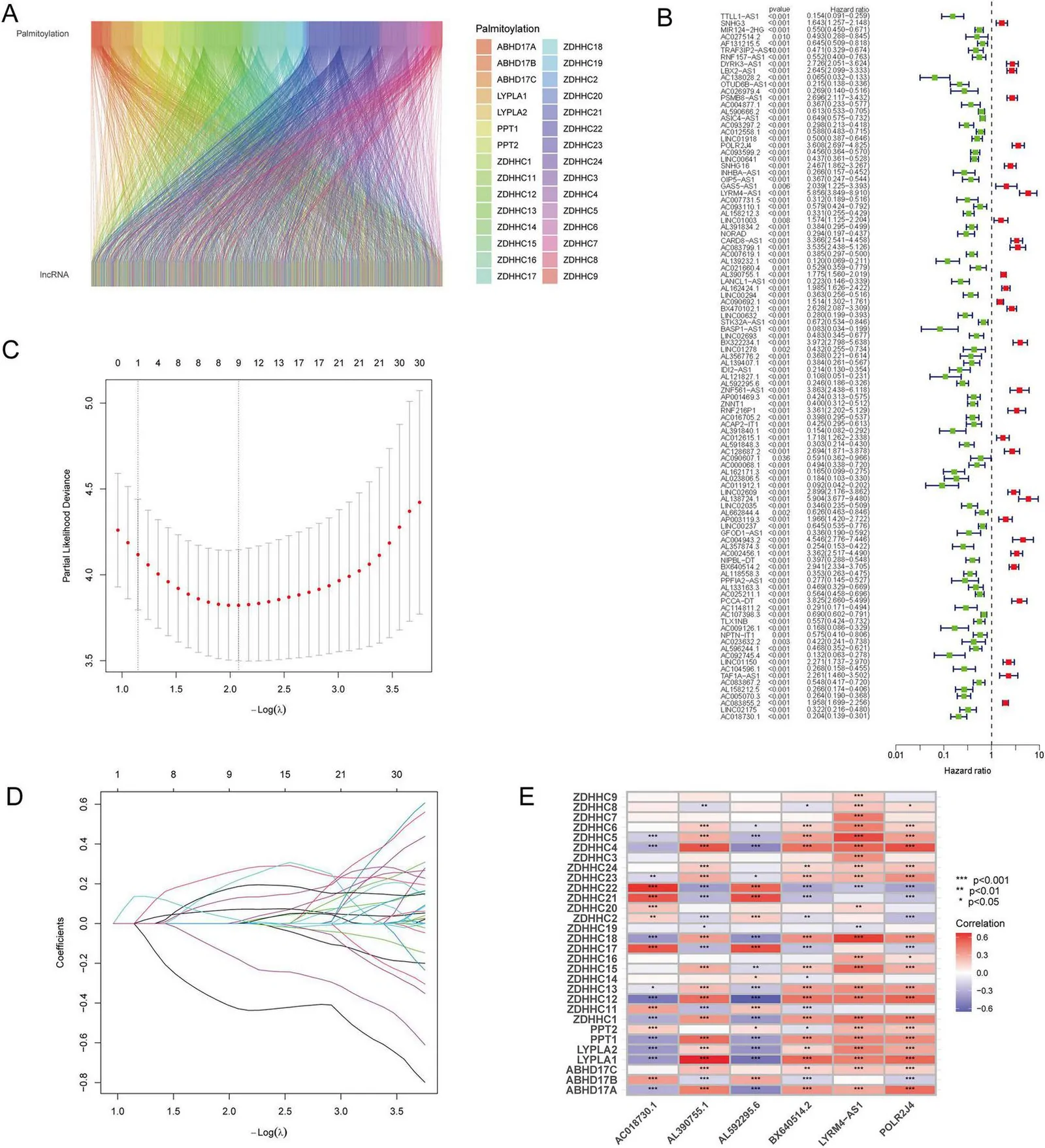
Identification of prognosis-related PRlncRNAs and construction of the risk signature. **(A)** Co-expression network between PRGs and lncRNAs. **(B)** Univariate Cox regression forest plot of survival-associated PRlncRNAs. **(C)** LASSO cross-validation curve. **(D)** LASSO coefficient profiles. **(E)** Correlation matrix between the six model PRlncRNAs and PRGs. *P < 0.05, **P < 0.01, and ***P < 0.001; ns, not significant.

The study workflow is shown in Figure 1. Using TCGA-Glioma transcriptomic data and 30 curated PRGs, we first performed co-expression analysis to identify PRG-associated lncRNAs. A total of 604 PRlncRNAs were identified (Figure 2A; Supplementary Table S3). Univariate Cox regression further identified 104 PRlncRNAs significantly associated with overall survival in glioma (Figure 2B). LASSO-Cox regression was then used to refine the prognostic variables and construct a stable risk model (Figures 2C,D). Multivariate Cox regression identified six PRlncRNAs in the final prognostic signature: POLR2J4, LYRM4-AS1, BX640514.2, AL592295.6, AL390755.1, and AC018730.1. Correlation analysis showed that these six model lncRNAs were closely associated with multiple PRGs (Figure 2E). The final risk-score formula was: risk score = POLR2J4 × 0.459 + LYRM4-AS1 × 0.535 + BX640514.2 × 0.259 + AL592295.6 × (-0.627) + AL390755.1 × 0.126 + AC018730.1 × (-0.468).

### 3.2 Prognostic validation of the PRlncRNA signature

**FIGURE 3:**
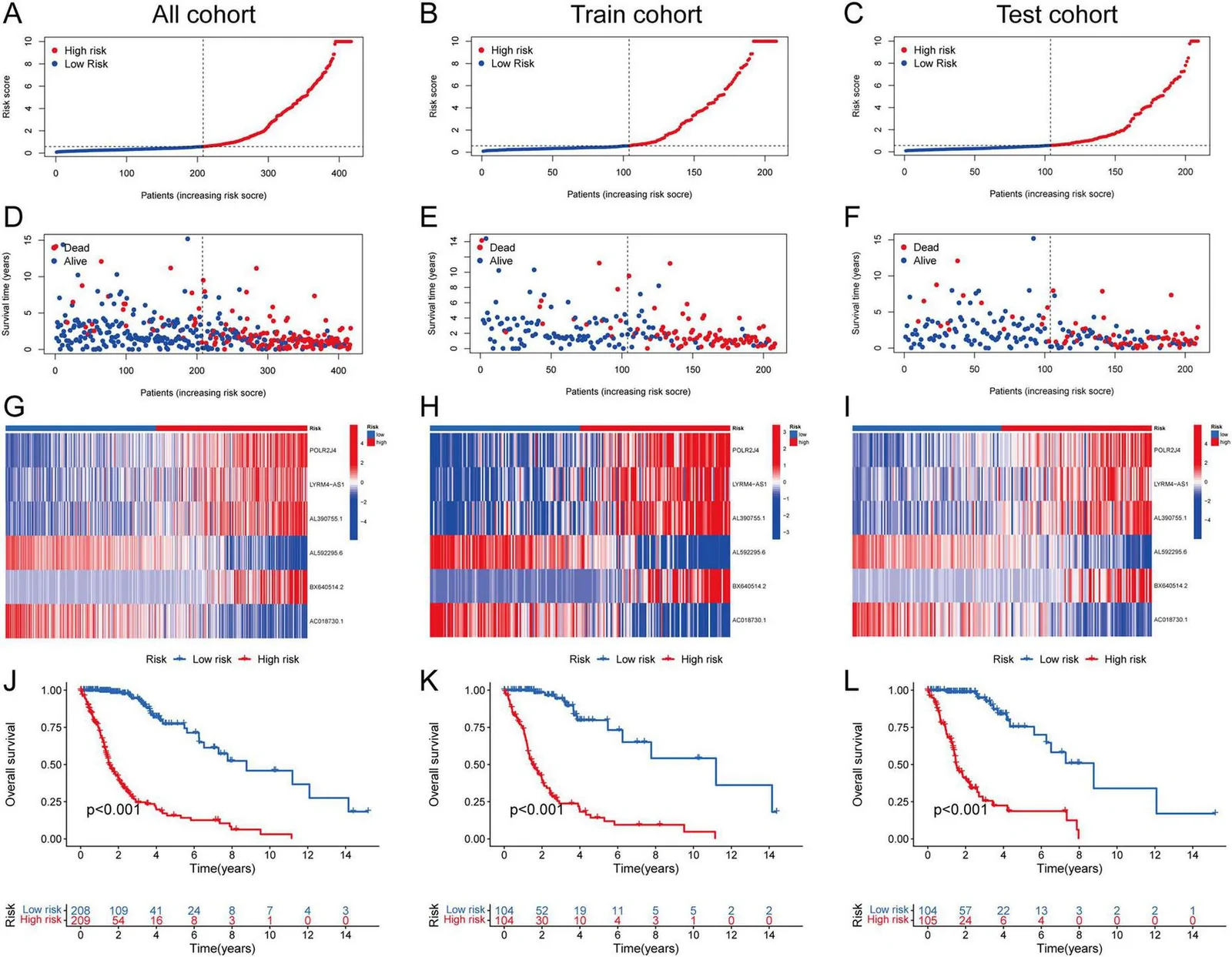
Prognostic validation of the PRlncRNA signature. **(A–C)** Risk-score distributions in the total, training, and validation cohorts. **(D–F)** Survival-status plots. **(G–I)** Heatmaps showing expression of the six model PRlncRNAs. **(J–L)** Kaplan-Meier survival curves for high- and low-risk groups.

Patients were stratified into high- and low-risk groups according to the median risk score. In the total TCGA-Glioma cohort, training cohort, and validation cohort, the risk-score distribution plots showed a progressive increase in risk score from low- to high-risk patients (Figures 3A–C). Survival-status plots demonstrated that death events increased and survival time decreased as the risk score increased (Figures 3D–F). Heatmaps showed distinct expression patterns of the six PRlncRNAs between the two risk groups (Figures 3G–I). Kaplan-Meier curves demonstrated that high-risk patients had significantly worse overall survival than low-risk patients in the total cohort, training cohort, and validation cohort (Figures 3J–L). These findings indicate that the PRlncRNA signature robustly stratifies glioma prognosis.

### 3.3 Association between the risk score and clinicopathological features

We next examined the relationship between the PRlncRNA risk score and clinicopathological variables. Heatmap analysis showed that age, tumor grade, IDH status, 1p/19q co-deletion status, and MGMT promoter methylation status were significantly different between high- and low-risk groups, whereas sex was not significantly different (Supplementary Figure S1A). Subgroup survival analyses further showed that high-risk patients had poorer overall survival across clinically relevant strata (Supplementary Figure S1B). These results suggest that the risk score captures prognostic information that is broadly applicable across glioma clinical and molecular subgroups.

### 3.4 Independent prognostic value and nomogram performance

**FIGURE 4:**
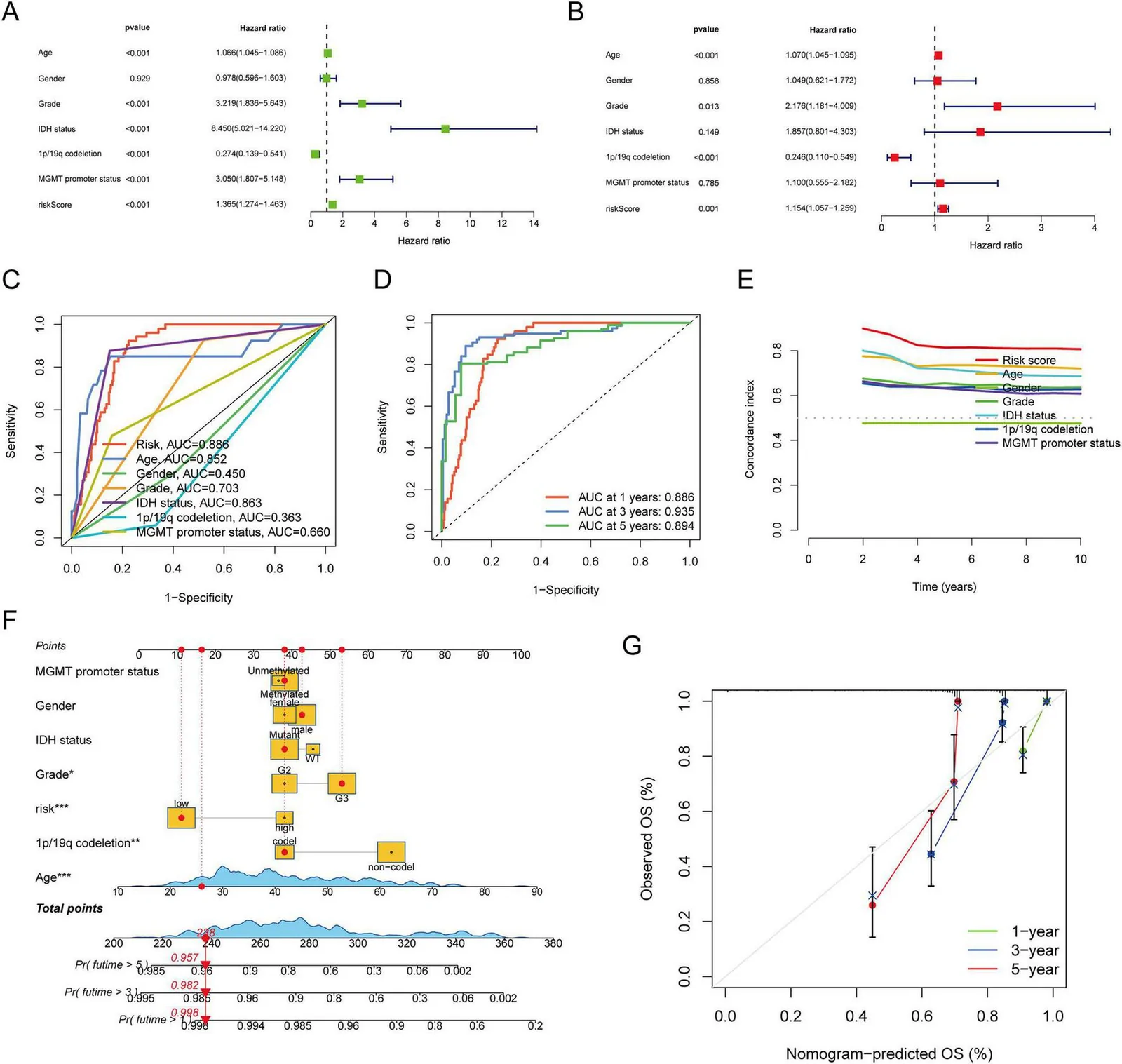
Independent prognostic analysis and nomogram construction. **(A)** Univariate Cox regression. **(B)** Multivariate Cox regression. **(C)** ROC curves comparing the risk score with clinicopathological variables. **(D)** Time-dependent ROC curves for 1-, 3-, and 5-year OS. **(E)** C-index comparison. **(F)** Nomogram for predicting 1-, 3-, and 5-year OS. **(G)** Calibration curves.

Univariate Cox regression showed that age, tumor grade, IDH status, 1p/19q co-deletion status, MGMT promoter methylation status, and the risk score were significantly associated with overall survival (Figure 4A). Among these variables, IDH status (HR = 8.450, 95% CI: 5.021–14.220) and the risk score (HR = 1.365, 95% CI: 1.274–1.463) showed prominent prognostic effects. Multivariate Cox regression demonstrated that age, tumor grade, 1p/19q co-deletion status, and the risk score were independent prognostic factors (Figure 4B). ROC analysis indicated that the risk score outperformed several single clinicopathological factors (Figure 4C). Time-dependent ROC analysis showed strong predictive accuracy for 1-, 3-, and 5-year overall survival, with AUC values of 0.886, 0.935, and 0.894, respectively (Figure 4D). C-index analysis further supported the superior prognostic performance of the risk score over time (Figure 4E). A nomogram integrating the risk score and clinical variables was constructed to estimate 1-, 3-, and 5-year survival probabilities (Figure 4F). Calibration curves showed good agreement between predicted and observed survival outcomes (Figure 4G).

### 3.5 Functional enrichment of risk-associated DEGs

**FIGURE 5:**
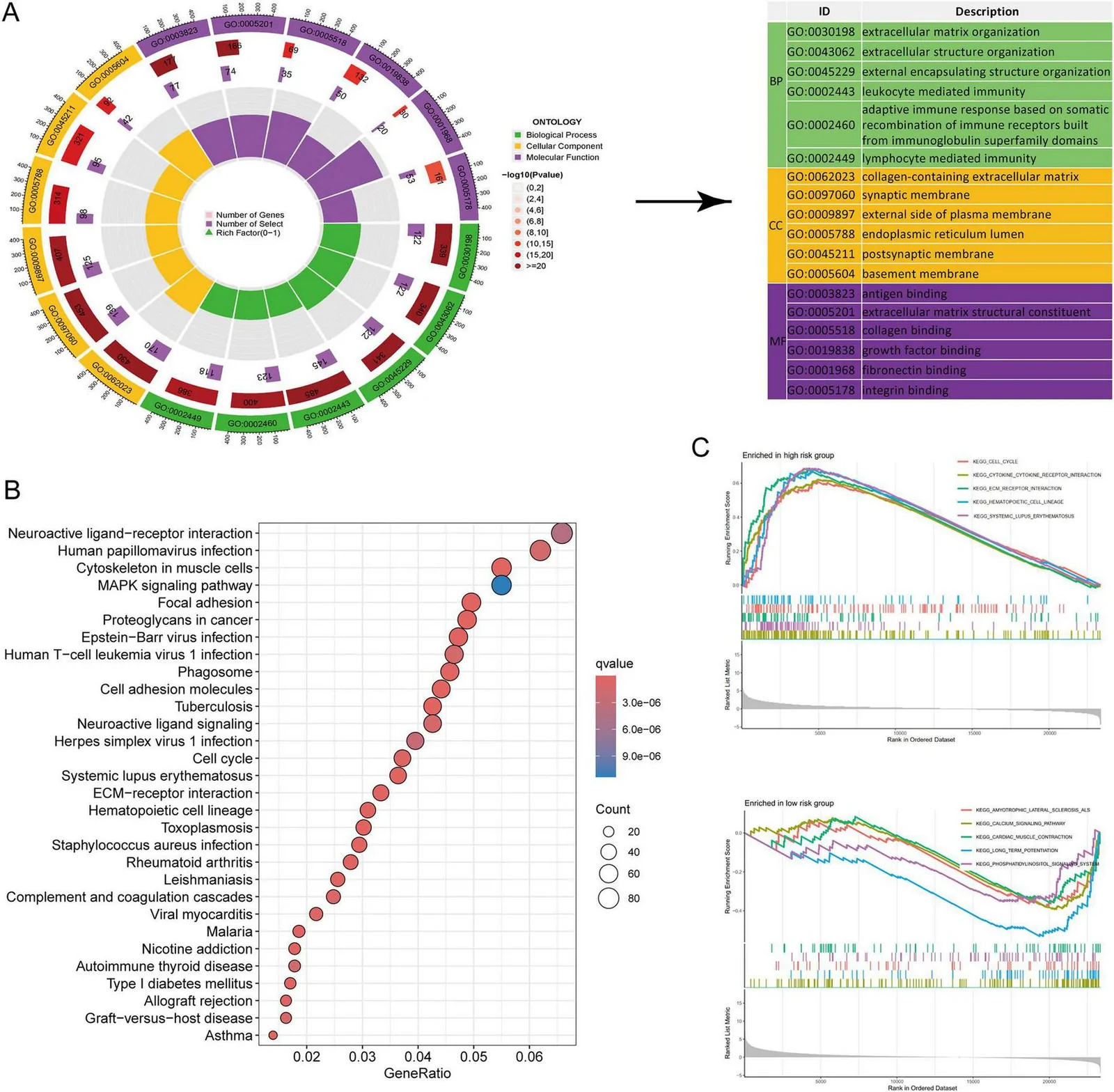
Functional enrichment analysis of risk-associated DEGs. **(A)** GO enrichment analysis. **(B)** KEGG pathway enrichment analysis. **(C)** GSEA showing pathways enriched in high- and low-risk groups.

A total of 1,088 DEGs were identified between high- and low-risk groups (Supplementary Table S4). GO enrichment analysis showed that these genes were mainly involved in extracellular matrix organization, leukocyte-mediated immunity, collagen-containing extracellular matrix, synaptic membrane, antigen binding, and extracellular matrix structural constituents (Figure 5A). KEGG enrichment analysis revealed enrichment of neuroactive ligand-receptor interaction, human papillomavirus infection, MAPK signaling, and other pathways (Figure 5B). Although some KEGG terms reflect broad pathway annotations, the enrichment of neuroactive signaling, ECM remodeling, and MAPK signaling suggests that the PRlncRNA risk score is linked to glioma-relevant molecular signaling and tumor-microenvironmental remodeling. GSEA further showed that high-risk tumors were enriched in cell-cycle programs, cytokine-cytokine receptor interaction, ECM-receptor interaction, and hematopoietic cell lineage pathways, whereas low-risk tumors were enriched in oxidative phosphorylation and cardiac muscle contraction-related gene sets (Figure 5C).

### 3.6 Immune infiltration and tumor microenvironment characteristics

CIBERSORT analysis showed that high-risk tumors tended to display increased infiltration of several immune-cell populations, including T-cell subsets, macrophage-related populations, neutrophils, and other immune regulatory components, whereas low-risk tumors showed relatively higher enrichment of selected antitumor immune populations, such as activated NK cells and monocytes (Supplementary Figure S2A). Consistently, ssGSEA analysis suggested that the high-risk group was enriched for multiple immune-cell and immune-function signatures, including dendritic cells, cytotoxic cells, macrophages, neutrophils, NK/T-cell-related signatures, and helper T-cell subsets (Supplementary Figure S2B). Additional deconvolution algorithms, including xCell, quanTIseq, TIMER, EPIC, and MCP-counter, further supported a broad association between the risk score and immune-cell infiltration patterns, and the detailed multi-algorithm comparisons are provided in Supplementary Figure S2C. High-risk tumors had significantly higher StromalScore, ImmuneScore, and ESTIMATEScore than low-risk tumors (Supplementary Figure S2D). Correlation analyses showed that the risk score was positively correlated with StromalScore (*R* = 0.633, *P* < 0.001), ImmuneScore (*R* = 0.564, *P* < 0.001), and ESTIMATEScore (*R* = 0.608, *P* < 0.001) (Supplementary Figure S2E). These data indicate that the high-risk group is characterized by stronger immune and stromal infiltration, which may reflect an immune-remodeled but suppressive glioma microenvironment.

### 3.7 Immunotherapy-response features, TMB, and MSI

**FIGURE 6:**
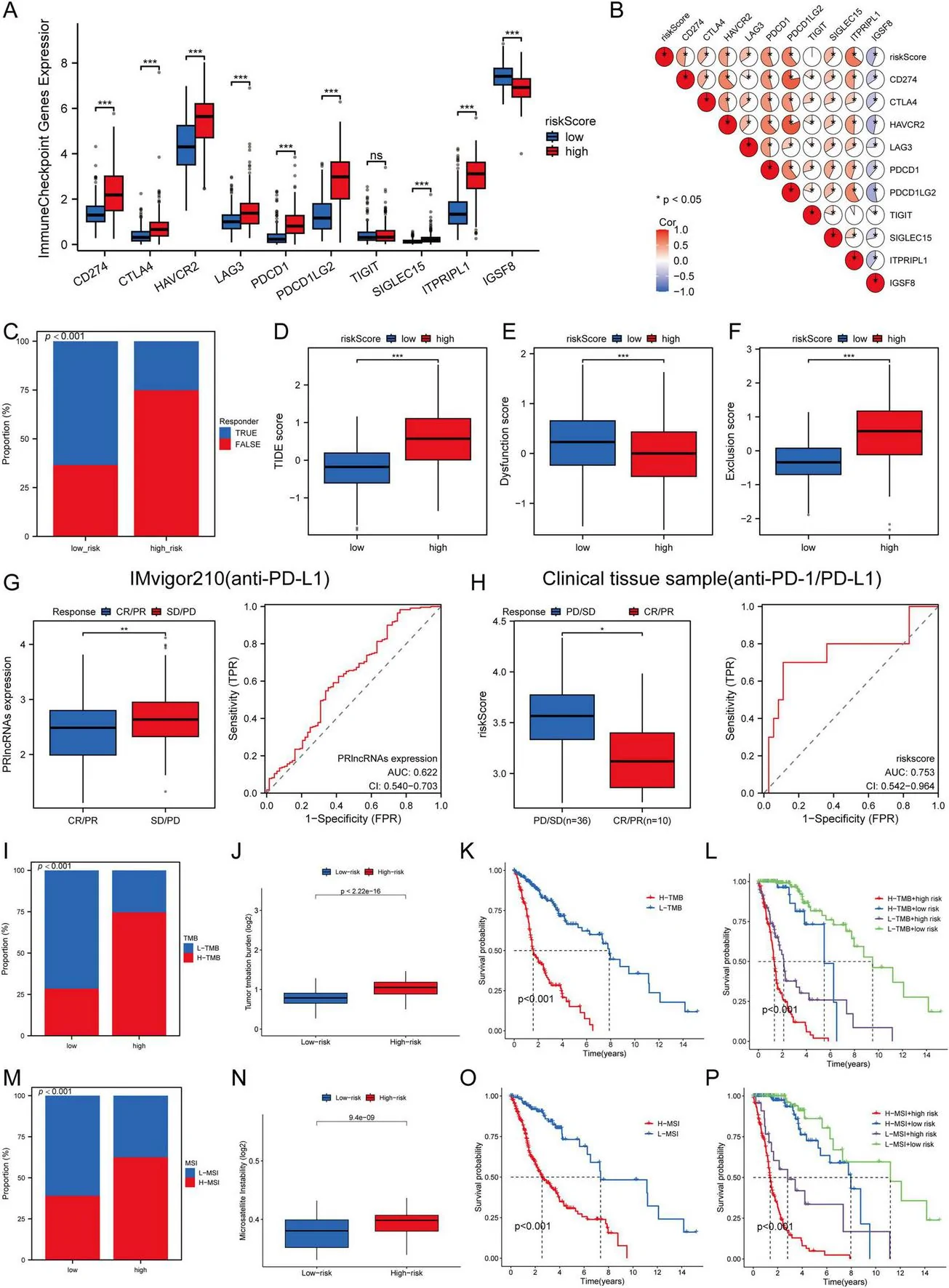
Immunotherapy-response, TMB, and MSI analyses. **(A)** Expression differences in immune checkpoint genes between high- and low-risk groups. **(B)** Correlation heatmap between the risk score and immune checkpoint genes. **(C)** Predicted immunotherapy responder proportions. **(D–F)** TIDE score, dysfunction score, and exclusion score. **(G)** IMvigor210 exploratory immunotherapy validation. **(H)** Clinical anti-PD-1/PD-L1 cohort validation. **(I,J)** TMB group distribution and TMB score comparison. **(K,L)** Survival analyses combining TMB and risk group. **(M,N)** MSI group distribution and MSI score comparison. **(O,P)** Survival analyses combining MSI and risk group. *P < 0.05, **P < 0.01, and ***P < 0.001; ns, not significant.

Immune checkpoint analysis showed that CD274, CTLA4, HAVCR2, LAG3, PDCD1, PDCD1LG2, SIGLEC15, ITPRIPL1, and IGSF8 were significantly upregulated in the high-risk group compared with the low-risk group, whereas TIGIT did not differ significantly (Figure 6A). Correlation analysis showed positive associations between the risk score and multiple immune checkpoint genes (Figure 6B). TIDE-based analysis indicated that the predicted immunotherapy responder proportion was higher in the low-risk group than in the high-risk group (Figure 6C). The high-risk group had higher TIDE score, dysfunction score, and exclusion score, suggesting greater immune escape potential (Figures 6D–F). In the IMvigor210 anti-PD-L1 cohort, a publicly available anti-PD-L1-treated urothelial carcinoma cohort with transcriptomic profiles and annotated clinical responses, PRlncRNA expression differed between responders and non-responders, and the model showed exploratory predictive value for immunotherapy response, with an AUC of 0.622 (Figure 6G). For further clinical validation, we analyzed an institutional self-collected anti-PD-1/PD-L1-treated cohort with available expression and response data; patients were classified as responders or non-responders according to the original clinical response records. In this cohort, non-responders showed significantly higher risk scores than responders, and the risk score predicted immunotherapy response with an AUC of 0.753 (Figure 6H). TMB analysis showed that the high-risk group had a higher proportion of high-TMB patients and significantly higher TMB scores (Figures 6I,J). Combined TMB-risk stratification showed that patients with high TMB and high risk had the poorest survival (Figures 6K,L). MSI analysis similarly showed higher MSI scores in the high-risk group, and patients with high MSI and high risk had the worst prognosis (Figures 6M–P).

### 3.8 Predicted drug-sensitivity differences between risk groups

Drug-sensitivity analysis was performed to identify potential therapeutic vulnerabilities associated with the PRlncRNA risk score. Compared with the low-risk group, the high-risk group showed significantly lower predicted IC50 values for 5-fluorouracil, alpelisib, buparlisib, camptothecin, cisplatin, crizotinib, dasatinib, entospletinib, gemcitabine, irinotecan, luminespib, pictilisib, rapamycin, ribociclib, savolitinib, talazoparib, taselisib, teniposide, topotecan, tozasertib, trametinib, and uprosertib (Supplementary Figure S3). These findings suggest that high-risk gliomas may have differential sensitivity to selected cytotoxic, targeted, and pathway-directed agents. However, as these results are based on transcriptome-guided pharmacogenomic prediction, they should be considered hypothesis-generating and require experimental validation.

### 3.9 Clinical and cellular validation of the six PRlncRNAs

**FIGURE 7:**
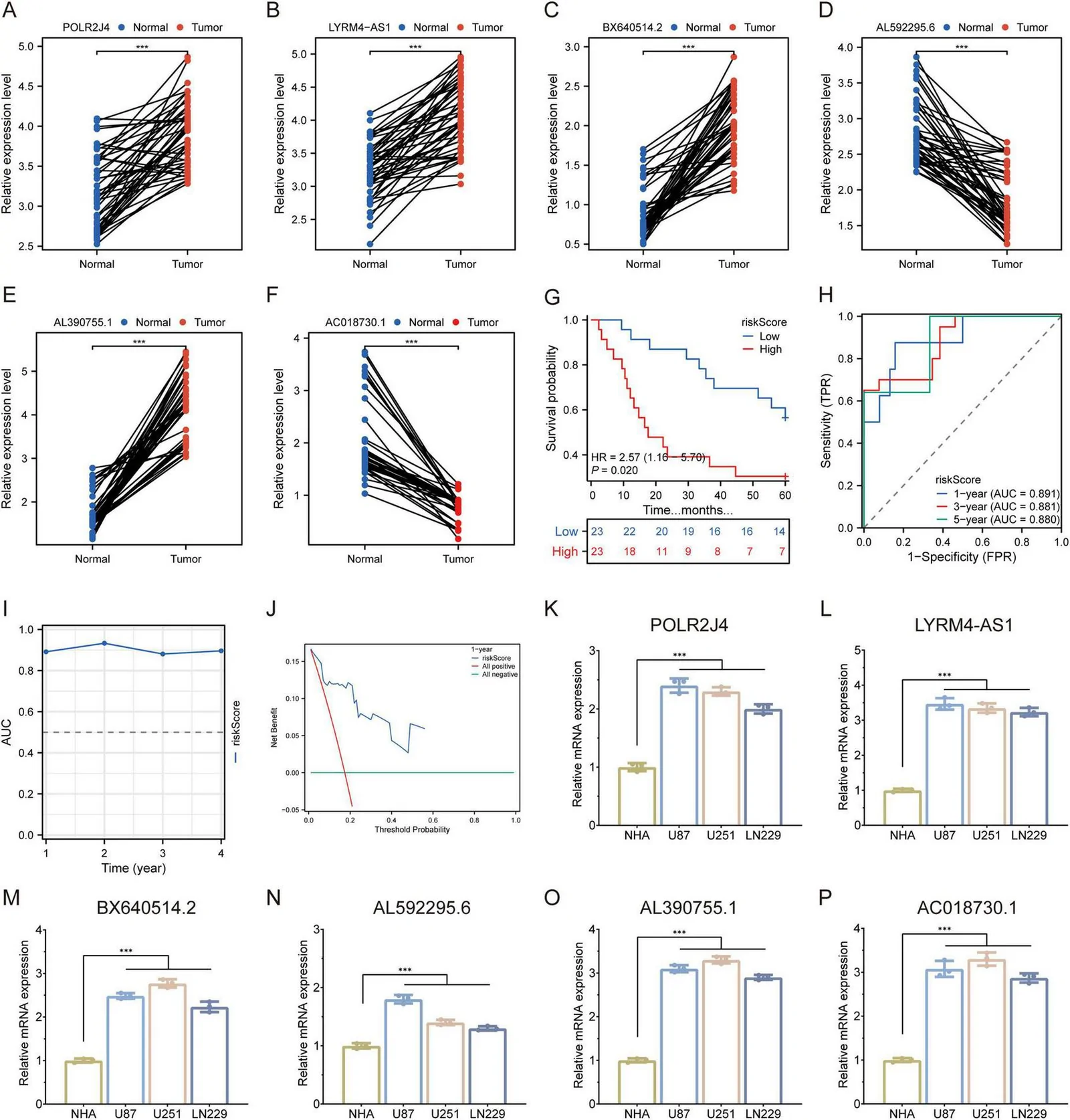
Clinical and cellular validation of the PRlncRNA signature. **(A–F)** qRT-PCR validation of POLR2J4, LYRM4-AS1, BX640514.2, AL592295.6, AL390755.1, and AC018730.1 in paired glioma and adjacent tissues. **(G)** Kaplan-Meier survival curve in the clinical validation cohort. **(H)** Time-dependent ROC curves. **(I)** Time-dependent AUC curve. **(J)** Decision curve analysis. **(K–P)** Expression of the six model PRlncRNAs in glioma cell lines and NHA cells. ***P < 0.001 and ****P < 0.0001.

qRT-PCR analysis of 46 paired clinical samples showed that POLR2J4, LYRM4-AS1, BX640514.2, and AL390755.1 were significantly upregulated in glioma tissues compared with adjacent tissues, whereas AL592295.6 and AC018730.1 were significantly downregulated (Figures 7A–F). Applying the TCGA-derived formula to the clinical validation cohort stratified patients into high- and low-risk groups. Kaplan-Meier analysis showed that high-risk patients had significantly poorer overall survival than low-risk patients (*P* = 0.020; HR = 2.57; 95% CI: 1.16–5.70) (Figure 7G). Time-dependent ROC analysis showed AUC values of 0.891, 0.881, and 0.880 for 1-, 3-, and 5-year survival prediction, respectively (Figure 7H), and the time-dependent AUC curve indicated stable predictive performance during follow-up (Figure 7I). DCA supported the clinical net benefit of the risk-score model within a reasonable threshold-probability range (Figure 7J). In cell lines, all six model lncRNAs showed differential expression in glioma cells compared with NHA cells, supporting their biological relevance in glioma (Figures 7K–P).

### 3.10 LYRM4-AS1 knockdown suppresses glioma cell proliferation, migration, invasion, and colony formation

**FIGURE 8:**
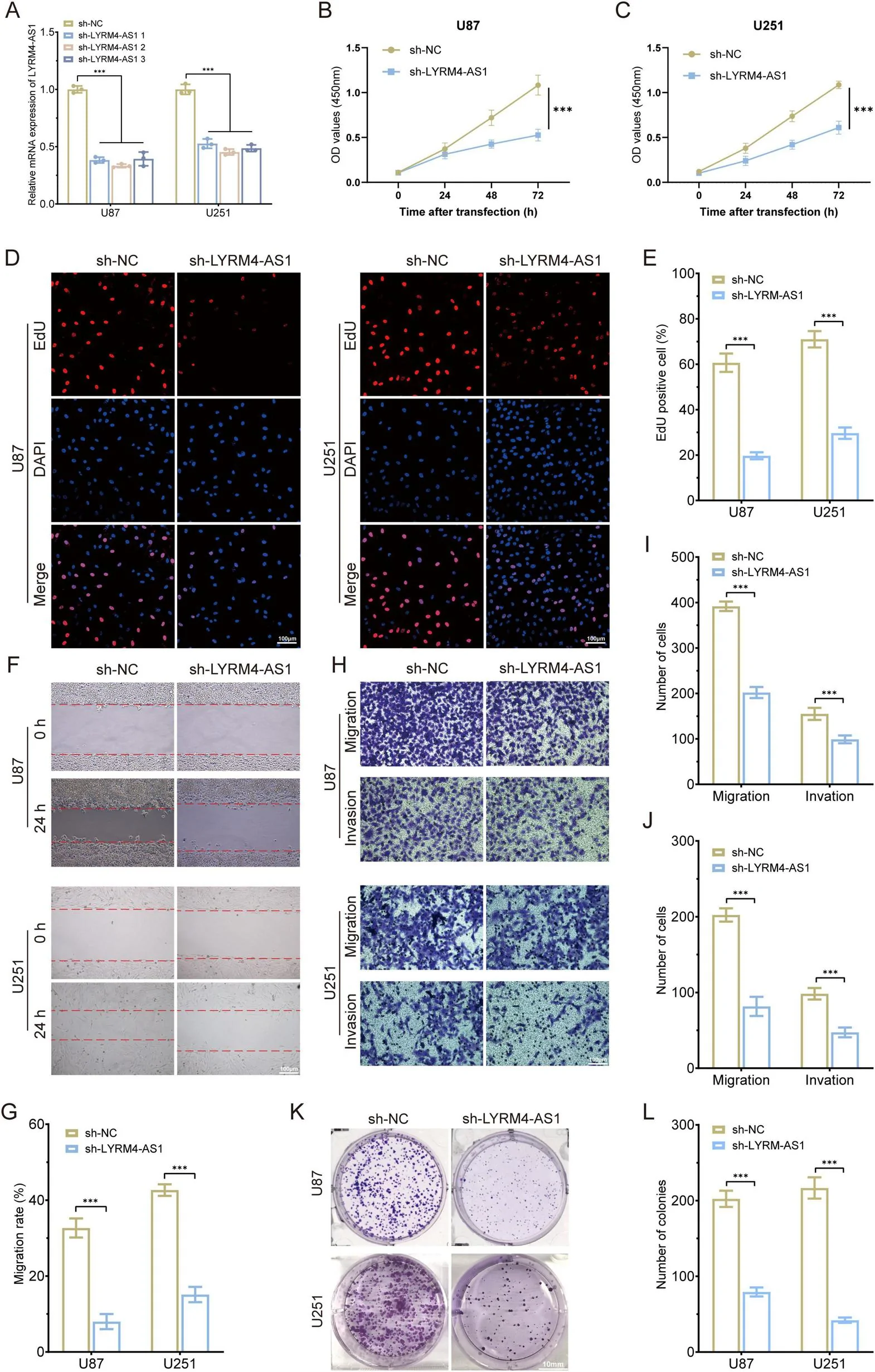
LYRM4-AS1 knockdown suppresses malignant phenotypes *in vitro*. **(A)** qRT-PCR analysis of LYRM4-AS1 knockdown efficiency. **(B,C)** CCK-8 assays in U87 and U251 cells. **(D,E)** EdU staining and quantification. **(F,G)** Wound-healing assays and quantification. **(H–J)** Transwell migration/invasion assays and quantification. **(K,L)** Colony-formation assays and quantification. ***P < 0.001 and ****P < 0.0001.

Because LYRM4-AS1 was a positive-risk component of the model and was consistently upregulated in glioma tissues and cell lines, it was selected for functional validation. Three independent shRNAs targeting LYRM4-AS1 were tested in U87 and U251 cells, and qRT-PCR confirmed efficient knockdown (Figure 8A). CCK-8 assays showed that LYRM4-AS1 knockdown significantly reduced cell viability over time in both U87 and U251 cells (Figures 8B,C). EdU staining demonstrated a marked decrease in EdU-positive cells after LYRM4-AS1 silencing, indicating impaired DNA synthesis and proliferation (Figures 8D,E). Wound-healing assays showed that LYRM4-AS1 knockdown significantly reduced migratory capacity (Figures 8F,G). Transwell assays further confirmed that LYRM4-AS1 silencing suppressed migration and invasion in both cell lines (Figures 8H–J). Colony-formation assays showed that LYRM4-AS1 knockdown significantly reduced clonogenic growth (Figures 8K,L). These findings indicate that LYRM4-AS1 promotes multiple malignant phenotypes in glioma cells.

### 3.11 LYRM4-AS1 knockdown suppresses xenograft growth and alters tumor histological markers

**FIGURE 9:**
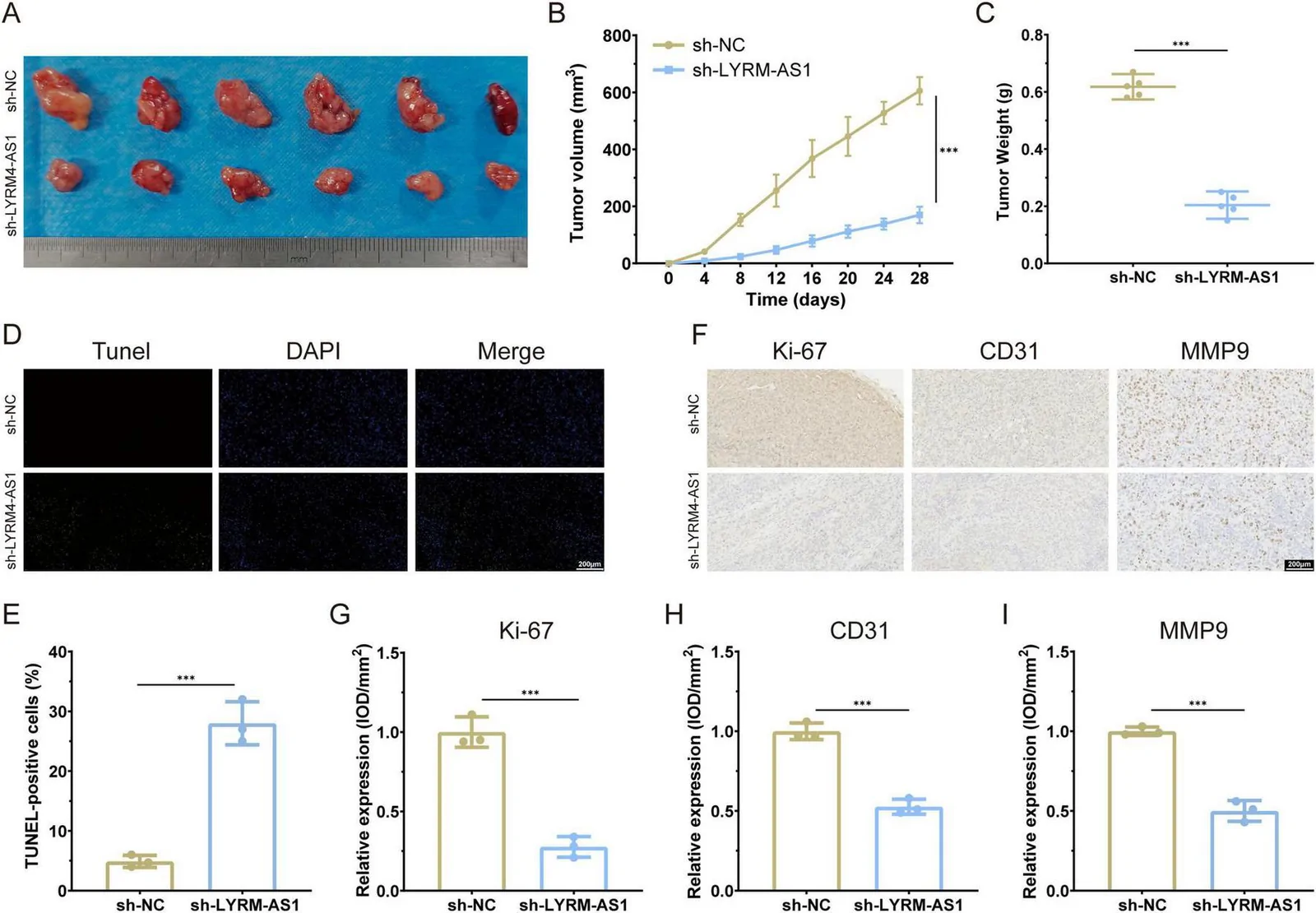
LYRM4-AS1 knockdown suppresses xenograft growth *in vivo*. **(A)** Representative xenograft tumors. **(B)** Tumor-volume curves. **(C)** Tumor weight. **(D,E)** TUNEL staining and quantification. **(F–I)** Immunohistochemistry and quantification of Ki-67, CD31, and MMP9. ***P < 0.001 and ****P < 0.0001.

The *in vivo* function of LYRM4-AS1 was evaluated using a nude-mouse xenograft model. Tumors derived from LYRM4-AS1-knockdown cells were visibly smaller than control tumors (Figure 9A). Quantitative analysis showed that LYRM4-AS1 knockdown significantly reduced tumor growth over time and decreased tumor weight at the endpoint (Figures 9B,C). TUNEL staining showed a higher proportion of apoptotic cells in sh-LYRM4-AS1 tumors than in sh-NC tumors (Figures 9D,E). Immunohistochemical analysis showed that Ki-67, CD31, and MMP9 expression levels were significantly reduced in LYRM4-AS1-knockdown tumors (Figures 9F–I), suggesting decreased proliferation, angiogenesis, and invasive potential. Together, these *in vivo* results support LYRM4-AS1 as a functional driver of glioma progression.

## 4 Discussion

This study systematically characterized palmitoylation-related lncRNAs in glioma and established a six-PRlncRNA signature with prognostic, immunological, and therapeutic relevance. The model stratified patients into survival-distinct risk groups in the TCGA-Glioma cohort and retained prognostic value in training, validation, and clinical cohorts. Importantly, the risk score remained an independent prognostic factor after adjustment for clinicopathological and molecular variables, including age, grade, IDH status, 1p/19q co-deletion status, and MGMT promoter methylation status. These findings suggest that PRlncRNAs may complement established glioma biomarkers and provide additional information about tumor signaling state and microenvironmental remodeling.

The biological rationale for a palmitoylation-related lncRNA model is supported by the dual role of palmitoylation in neural signaling and cancer biology. In neurons, palmitoylation regulates synaptic receptor localization, membrane trafficking, and activity-dependent plasticity. In tumors, altered palmitoylation can influence oncogenic signaling, immune regulation, and therapy response. Thus, palmitoylation-related transcriptional states may be particularly relevant in glioma, a tumor type that arises in the nervous system and frequently exploits neural developmental and inflammatory programs. Our model did not directly measure palmitoylation at the protein level; rather, it used co-expression with curated PRGs to identify lncRNAs associated with palmitoylation-related transcriptional programs. This distinction is important and indicates that future studies should integrate lipidomics, palmitoyl-proteomics, acyl-biotin exchange assays, and perturbation of palmitoyltransferases to define direct mechanistic relationships.

The risk score was strongly associated with pathways involved in extracellular matrix organization, leukocyte-mediated immunity, neuroactive ligand-receptor interaction, MAPK signaling, cytokine-receptor interaction, and ECM-receptor interaction. These pathways are relevant to glioma because tumor progression depends not only on intrinsic proliferation programs but also on interactions with neural, vascular, stromal, and immune compartments. The enrichment of neuroactive ligand-receptor interaction is particularly consistent with the neural-tumor context, whereas enrichment of ECM and cytokine pathways suggests that the signature reflects microenvironmental communication. The association with MAPK signaling further supports the molecular signaling orientation of the model and fits the scope of the Molecular Signaling and Pathways section.

A key finding of this study is that high-risk tumors had higher StromalScore, ImmuneScore, and ESTIMATEScore and showed positive correlations between the risk score and tumor microenvironment scores. In many cancers, high immune infiltration may indicate an active antitumor immune state; however, in glioma, immune infiltration often reflects abundant tumor-associated microglia/macrophages, suppressive myeloid cells, and chronic inflammatory signaling. Therefore, the high-risk immune-enriched phenotype observed here should not be interpreted simply as a beneficial immune-active state. Instead, it likely represents an immune-remodeled and immunosuppressive microenvironment. This interpretation is supported by the higher expression of multiple immune checkpoint genes and higher TIDE, dysfunction, and exclusion scores in the high-risk group.

The immunotherapy-related results provide an additional translational dimension. Although low-risk patients showed a higher predicted responder proportion, high-risk patients had higher expression of several checkpoint genes and higher TMB/MSI scores. Notably, TIGIT did not differ significantly between the two risk groups, suggesting that not all immune checkpoints followed the same risk-associated expression pattern and that checkpoint remodeling in this model may be selective. This apparent contrast is biologically plausible: checkpoint expression and mutation burden alone do not guarantee effective antitumor immunity, particularly in glioma, where blood-brain barrier features, myeloid-dominant infiltration, T-cell exhaustion, and spatial exclusion can limit immune checkpoint blockade. Checkpoint blockade has transformed cancer therapy in several tumor types by reactivating antitumor immunity (Ribas and Wolchok, 2018). However, glioblastoma trials using PD-1 blockade have reported limited overall benefit in unselected patients (Havel et al., 2019). Pan-cancer analyses have shown that immunotherapy biomarkers are strongly influenced by tumor lineage and immune context (Samstein et al., 2019). TMB can enrich for immunotherapy response in selected settings, but its predictive value varies across tumor types (Marabelle et al., 2020). In glioma, mismatch repair deficiency and treatment-induced hypermutation further complicate the interpretation of mutation burden (Reardon et al., 2020). Combination strategies that address myeloid suppression, immune exclusion, or local brain-tumor barriers may therefore be required for effective immunotherapy (Cloughesy et al., 2019). Neoantigen vaccination has also generated intratumoral T-cell responses in glioblastoma, indicating that immune activation remains feasible in selected molecular contexts (Keskin et al., 2019). A recent cuproptosis-associated PDHA1 study further showed that pathway-centered multi-omics and clinical validation can identify immune-oncogenic axes, including E2F1-PD-L1 signaling, with relevance to immunotherapy responsiveness (Qin et al., 2026a). In this study, the clinical anti-PD-1/PD-L1 validation cohort supported the association between lower risk score and better response, but the sample size remains limited. Prospective glioma-specific immunotherapy cohorts will be required to determine the true predictive value of the PRlncRNA signature.

The TMB/MSI analyses suggest that the PRlncRNA risk score may provide information beyond genomic instability alone. Patients with high TMB and high risk, as well as those with high MSI and high risk, had the poorest survival. In pan-cancer studies, TMB has been associated with immunotherapy benefit in selected tumors (Samstein et al., 2019). However, its performance varies by tumor type and immune context (Marabelle et al., 2020). Gliomas have additional complexity because mismatch repair deficiency, temozolomide-induced hypermutation, immune exclusion, and myeloid-dominant inflammation can modulate the relationship between mutation burden and immune response (Reardon et al., 2020). Integrating PRlncRNA-defined signaling and microenvironmental states with TMB/MSI may therefore improve risk stratification compared with interpreting mutation burden alone.

Clinical and cellular validation strengthened the reliability of the signature. In 46 paired clinical samples, POLR2J4, LYRM4-AS1, BX640514.2, and AL390755.1 were upregulated in glioma tissues, whereas AL592295.6 and AC018730.1 were downregulated. The clinical cohort also confirmed that the risk score stratified overall survival and showed strong time-dependent predictive accuracy. Among the model lncRNAs, LYRM4-AS1 was selected for functional validation because it was a positive-risk component and showed consistent upregulation. LYRM4-AS1 knockdown suppressed glioma cell proliferation, DNA synthesis, migration, invasion, and colony formation *in vitro*. *In vivo*, LYRM4-AS1 silencing inhibited xenograft growth, increased TUNEL-positive apoptosis, and reduced Ki-67, CD31, and MMP9 expression. These data indicate that LYRM4-AS1 is not merely a prognostic marker but a functional contributor to glioma progression.

The mechanistic role of LYRM4-AS1 remains to be defined. The reduction of Ki-67 suggests impaired proliferation; decreased CD31 indicates reduced angiogenic activity; and reduced MMP9 suggests attenuation of matrix remodeling and invasive potential. These phenotypes are consistent with the enrichment of ECM-related, cytokine-related, and immune-related pathways in the high-risk group. However, this study did not determine whether LYRM4-AS1 regulates palmitoylation-related signaling directly or indirectly. Future studies should investigate LYRM4-AS1 subcellular localization, RNA-binding proteins, ceRNA networks, chromatin-associated effects, and interactions with palmitoylation enzymes or palmitoylated signaling proteins. Rescue experiments and pathway inhibition studies will also be needed to determine whether LYRM4-AS1 controls glioma progression through MAPK, PI3K/AKT, NF-kappaB, or other neuro-oncology-relevant pathways.

Several limitations should be acknowledged. First, the PRlncRNA signature was primarily derived from retrospective public datasets, and larger multicenter prospective validation is needed. Second, TCGA-Glioma includes molecularly diverse glioma subtypes, and subtype-specific performance should be evaluated in larger cohorts. Third, TIDE, TMB/MSI, and drug-sensitivity analyses are computational predictions and require independent clinical and experimental validation. Fourth, the IMvigor210 cohort is not glioma-specific and was used only as an exploratory immunotherapy dataset. Fifth, although LYRM4-AS1 was functionally validated, the direct molecular mechanism linking LYRM4-AS1 to palmitoylation-related signaling remains unresolved.

Overall, this study provides an integrated palmitoylation-related lncRNA framework for glioma. The six-PRlncRNA signature predicts prognosis, reflects molecular signaling and immune remodeling, and suggests therapeutic vulnerabilities. LYRM4-AS1 is validated as a functional driver of glioma progression and represents a candidate target for future mechanistic and translational studies.

## 5 Conclusion

We developed and validated a six-lncRNA palmitoylation-related signature that predicts prognosis and reflects immune and therapeutic features in glioma. The risk score was independently associated with overall survival and correlated with extracellular matrix remodeling, neuroactive signaling, immune infiltration, immune-checkpoint expression, TIDE scores, TMB/MSI status, and predicted drug sensitivity. Clinical validation confirmed the prognostic value of the model. Functional experiments demonstrated that LYRM4-AS1 knockdown suppresses glioma cell malignant phenotypes and xenograft growth. These findings identify PRlncRNAs as biomarkers at the intersection of neural tumorigenesis, molecular signaling, and immune remodeling, and nominate LYRM4-AS1 as a candidate functional target in glioma.

## Data Availability

The original contributions presented in the study are included in the article/Supplementary material, further inquiries can be directed to the corresponding authors.

## Supplementary material

Supplementary Table S1 Curated palmitoylation-related genes used in this study.

Supplementary Table S2 Clinicopathological information of TCGA-Glioma patients.

Supplementary Table S3 Palmitoylation-related lncRNAs identified by PRG-lncRNA co-expression analysis.

Supplementary Table S4 Differentially expressed genes between the high- and low-risk groups.
