# Supplementary material for: Palmitoylation-related lncRNAs link molecular signaling, immune remodeling, and tumor progression in glioma: prognostic modeling and functional validation of LYRM4-AS1
Source: Front Mol Neurosci. 2026 Sep 10;19:1901839. doi: 10.3389/fnmol.2026.1901839 (PMC13601299; doi:10.3389/fnmol.2026.1901839)
Supplement: Supplementary file 1 [file Data_Sheet_1.zip › Supplementary Figure.docx]

Supplementary Figure S1. Association between the risk score and clinicopathological features. (A) Heatmap showing clinical feature distributions between high- and low-risk groups. (B) Subgroup Kaplan-Meier survival analyses.

Supplementary Figure S2. Relationship between the risk score and immune infiltration. (A) CIBERSORT immune-cell comparisons. (B) ssGSEA immune-cell and immune-function comparisons. (C) Multi-algorithm immune correlation analysis. (D) StromalScore, ImmuneScore, and ESTIMATEScore comparisons. (E) Correlations between the risk score and tumor microenvironment scores.

Supplementary Figure S3. Drug-sensitivity prediction. Predicted IC50 differences for candidate compounds between high- and low-risk groups.
